# Dramatic pressure-driven enhancement of bulk skyrmion stability

**DOI:** 10.1038/srep21347

**Published:** 2016-02-19

**Authors:** I. Levatić, P. Popčević, V. Šurija, A. Kruchkov, H. Berger, A. Magrez, J. S. White, H. M. Rønnow, I. Živković

**Affiliations:** 1 Institute of Physics, Bijenička 46, HR-10 000, Zagreb, Croatia; 2 Institute of Solid State Physics, TU Wien, A-1040 Vienna, Austria; 3 Laboratory for Quantum Magnetism, Institute of Physics, EPFL, CH-1015 Lausanne, Switzerland; 4 Crystal Growth Facility, Institute of Physics, EPFL, CH-1015 Lausanne, Switzerland; 5 Laboratory for Neutron Scattering and Imaging, Paul Scherrer Institut, CH-5232 Villigen, Switzerland

## Abstract

The recent discovery of magnetic skyrmion lattices initiated a surge of interest in the scientific community. Several novel phenomena have been shown to emerge from the interaction of conducting electrons with the skyrmion lattice, such as a topological Hall-effect and a spin-transfer torque at ultra-low current densities. In the insulating compound Cu_2_OSeO_3_, magneto-electric coupling enables control of the skyrmion lattice via electric fields, promising a dissipation-less route towards novel spintronic devices. One of the outstanding fundamental issues is related to the thermodynamic stability of the skyrmion lattice. To date, the skyrmion lattice in bulk materials has been found only in a narrow temperature region just below the order-disorder transition. If this narrow stability is unavoidable, it would severely limit applications. Here we present the discovery that applying just moderate pressure on Cu_2_OSeO_3_ substantially increases the absolute size of the skyrmion pocket. This insight demonstrates directly that tuning the electronic structure can lead to a significant enhancement of the skyrmion lattice stability. We interpret the discovery by extending the previously employed Ginzburg-Landau approach and conclude that change in the anisotropy is the main driver for control of the size of the skyrmion pocket.

Skyrmions were first introduced in the context of topologically protected excitations in the field theory of hadrons^1^. Although later the quark theory proved successful, the concept of skyrmions and their mathematical beauty have found applications in various topics within many body physics, such as 2D electron gases^2^,^3^, Bose condensation^4^,^5^,^6^ and, particularly, magnetic systems. Initially, magnetic skyrmions were only theoretically predicted^7^,^8^, but subsequently they have been found experimentally in the form of skyrmion lattices in several chiral systems, including conductive MnSi^9^ and insulating 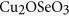
10. On top of the manipulation of the whole skyrmion lattice^11^,^12^,^13^, successful control of individual skyrmions has been demonstrated using lasers^14^ and scanning-tunneling microscopes^15^, indicating ‘skyrmionics’ to be a promising avenue for the field of spintronics.

The compounds that exhibit a skyrmion lattice display remarkably universal phase diagrams-see for example Fig. 1a. Below a critical temperature *T*_*C*_ the system orders as a helimagnet, with spins rotating within a plane that is perpendicular to the propagation vector 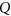, Fig. 1b. Upon the application of a magnetic field *B > B*_*C1*_ the magnetic structure transforms into a conical arrangement, Fig. 1c, where spins precess about a cone that is aligned with the direction of magnetic field. For even larger values *B > B*_*C2*_ the spins are field-polarized. The skyrmion phase is found to lie adjacent to the order-disorder transition, at approximately half of the value of the critical magnetic field *B ≈ B*_*C2*_/2. It exhibits a hexagonal arrangement^9^ of individual skyrmions, Fig. 1d, where the central spins point in the opposite direction to the applied magnetic field, and a chiral sense of rotation of the magnetic spins yields a finite topological charge^16^.

So far the temperature width of the skyrmion pocket in bulk samples has been limited to 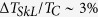 in all investigated cubic chiral compounds. A substantial enhancement of the size of the skyrmion pocket has been demonstrated for thin films and ultra thin slabs of these materials^10^,^17^, with thickness 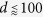 nm. In these quasi-2D systems the anisotropy is considered to play a major role in the enhanced stabilization of the skyrmion lattice^18^. On the other hand, the resultant phase diagrams are not generic and depend on the material as well as on the method of preparation^19^,^20^. Thus, finding a link between the universal phase diagram in bulk materials and the magneto-crystalline anisotropy presents a major challenge for the deeper understanding of the formation and stability of the skyrmion phase.

In bulk samples the anisotropy 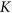 arises in the form of the spin-orbit interaction, and it is the weakest of the energy scales present in skyrmion compounds *K* ≪ *D* ≪ *J*, where 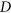 and 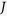 are the Dzyaloshinskii-Moriya and the ferromagnetic exchange interactions, respectively. 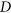 favors a perpendicular orientation of neighboring spins, and is responsible for the formation of the chiral helimagnet ground state with a long wavelength modulation *λ* ∼ *J*/D >> *a*, 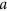 being the inter-atomic distance. The anisotropy 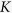 then pins the helices along preferred directions, and hence determines the value of the first critical magnetic field 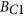 above which the conical structure is stabilized.

To further our understanding of the stability of the skyrmion lattice, it is imperative to establish the parameter range in which the skyrmion lattice is physically favorable with respect to other phases, and further how the size of the skyrmion phase depends on 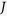, 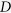 and 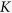. In that context, the application of hydrostatic pressure is a well-known technique which allows fine-tuning of energy scales through tiny shifts in atomic positions. Before the discovery of its skyrmion lattice phase, MnSi was intensively investigated^21^,^22^ due to a complete suppression of long-range magnetic order above a critical pressure (*p*_c _∼ 1.5 GPa) and the discovery of non-Fermi liquid behavior for 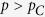. A similar suppression of magnetic order has been observed in FeGe^23^ around 19 GPa. In contrast, the ordering temperature of 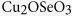 increases under pressure^24^,^25^, thus emphasizing the importance of detailed investigation of its pressure dependence. Here we present an extensive study of the phase diagram of 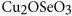 under hydrostatic pressure up to 2.3 GPa, and discover that the absolute size of the skyrmion pocket in bulk samples can be dramatically enlarged.

## Results

**Figure 1:**
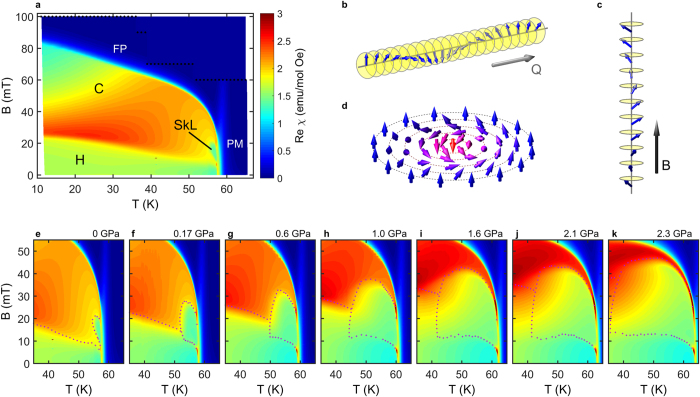
Development of the phase diagram of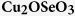 with pressure. (**a**) The phase diagram of 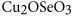 given by the real part of the magnetic susceptibility 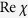 taken while ramping magnetic field down. Black dots mark the upper boundary of scans. It consists of H-helical phase, C-conical phase, SkL-skyrmion lattice, FP-field polarized phase, PM-paramagnet. (**b**) Helical spin arrangement. Magnetic moments rotate within the plane that is perpendicular to the direction of the wave vector 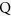. (**c**) Conical spin arrangement. The precession of magnetic moments outlines the cone which is oriented along the direction of magnetic field 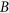. (**d**) Spin configuration of a single skyrmion. (**e**–**g**): Pressure dependence of the phase diagram around the skyrmion pocket. Pink dotted lines outline the phase boundaries between the helical, conical and skyrmion phases as determined by the imaginary component of the magnetic susceptibility.

High-quality magnetic ac susceptibility 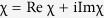 accurately traces the magnetic phases, as illustrated in Fig. 1a for zero-pressure. As can be seen from the zoomed-in part in Fig. 1e, the skyrmion phase is manifested as a region of lower susceptibility (yellow) compared to the surrounding conical phase (red). For 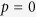 it occupies a very small part of the phase diagram, adjacent to the order-disorder boundary, with a maximum extent in temperature of 2 K. The maximum field range amounts to around 15 mT, and at lower field values the skyrmion pocket becomes narrower, forming a shape of an inverted tear-drop. The situation drastically changes by the application of even a small hydrostatic pressure (*p = *0.17 GPa, Fig. 1f). The skyrmion pocket increases in size, especially the low magnetic field region, more than doubling over the zero-pressure extent. In addition to the observed growth, one can notice a qualitative change in the way the phases are arranged in that part of the phase diagram. Namely, under pressure the skyrmion phase borders directly with the helimagnetic phase without the conical phase in-between. As shown in Fig. 1g–k, further increase of the pressure evidences a continuation of the growth of the low-susceptibility region, both towards lower temperatures as well as towards larger magnetic fields.

**Figure 2:**
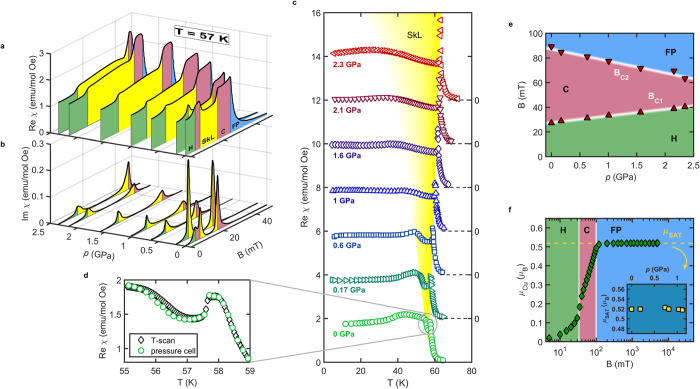
Evolution of magnetic phases with pressure. (**a**) real part and (**b**) imaginary part of the magnetic susceptibility 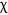 at *T *= 57 K. (**c**) Extracted temperature profiles through the middle of the skyrmion pocket at magnetic fields 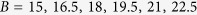 and 24 mT (from 0 to 2.3 GPa). (**d**) Comparison of the extracted temperature profile with a temperature scan taken in cooling measured on the same sample using a standard setup (without the pressure cell) at zero pressure and *B* = 15 mT. A small difference is visible on the low temperature side of the skyrmion pocket (≤0.2 K) where disintegration of the skyrmion lattice occurs and the exact path through the phase diagram becomes important. (**e**) Pressure dependence of the helical-conical and conical-field polarized metamagnetic phase transitions in the *T* = 0 K limit. (**f**) Magnetic field dependence of magnetization at *p * = 1.0 GPa and *T * = 10 K. Inset: Pressure dependence of the saturation magnetization level.

Quantitatively, we can identify the phase boundaries and extract their pressure dependence from individual scans. Fig. 2a,b show field scans of real and imaginary susceptibility at *T *= 57 K, the middle of the skyrmion pocket at zero pressure. The phase boundaries are determined by maxima in the imaginary component, except for the transition between the conical (red) and the field polarized state (blue) where a kink in the real part marks the boundary. On the high-field side the skyrmion pocket (yellow) transforms into the conical phase by a steep increase of the real component, while the imaginary part exhibits a sharp peak. On the low-field side the presence of the conical phase between the helimagnetic (green) and the skyrmion phase at zero pressure is revealed by two peaks in the imaginary part, while at elevated pressures a single peak is observed. To our knowledge this represents the first experimental evidence that the helimagnetic phase and the skyrmion phase are thermodynamically distinct. In the real part this transition becomes less pronounced, because *T*_*C*_ increases with pressure, placing field scans at 57 K further away from the ordering temperature. We showed recently that the strength of the susceptibility anomaly marking the border of the skyrmion pocket becomes quickly suppressed for 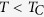
26.

In our experiment the temperature was slowly increased while magnetic field was continuously ramped between zero and a maximum value. Due to the slow temperature change (*dT/dt < 100μ*K/s) and reduced field range around the skyrmion pocket (black dots in Fig. 1a), successive field scans were spaced by no more than 0.2 K, providing a very good resolution in temperature. This enables us to determine the temperature width of the skyrmion phase by plotting 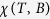 for fixed 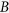. Extracted temperature profiles through the middle of the skyrmion pocket are displayed in Fig. 2c. In Fig. 2d we compare 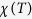 extracted from the 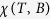 maps to a temperature scan recorded on cooling using a standard susceptibility setup. The overlap of the two curves demonstrates that the extracted profiles presented in Fig. 2c probe directly the thermodynamic phase boundaries.

Even at the smallest applied pressures an enhanced stability of the skyrmion phase is readily observed: compared to the zero pressure case, where 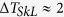 K (3% of 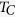), it reaches 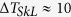 K at 0.6 GPa, corresponding to 17% of 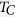. If the same relative increase is achieved in the recently discovered skyrmion compound^27^ Co_8_Zn_9_Mn_3_ with the ordering temperature at 322 K (49 °C), the skyrmion lattice would be stable even below 0 °C, this covering the usual operational temperature range for most electronic circuits.

With further increase of pressure the low-temperature boundary to the conical phase becomes less pronounced, transforming into a wide cross-over. At the highest pressure (*p *= 2.3 GPa) the low susceptibility region extends almost to 30 K, indicating that skyrmions exist down to at least half of the ordered phase diagram in the high-magnetic field region. It remains an open question as to what kind of a spatial distribution is taken by skyrmions at high pressures and whether they still form a well-defined lattice.

At low temperatures the helical and conical phases are thermodynamically stable, although with pressure the area covered by the conical phase is substantially reduced from both high and low field sides compared with ambient pressure, as revealed in Fig. 2e. More importantly, we observe an increase of the first critical magnetic field 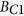 that marks the transition from the helical to the conical phase. As has been introduced above, 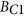 is linked directly to the anisotropy energy 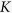 which determines the direction of the helical propagation vector in the absence of an applied magnetic field 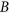. Through the adaptation of the effective model developed in ref.^9^, below we demonstrate that such an increase of 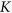 can explain the observed expansion of the bulk skyrmion phase stability.

We start from the usual Ginzburg-Landau functional for the magnetization 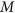 of the system^28^


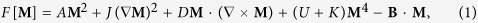


where the 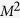 term is related to the temperature-dependent tuning parameter 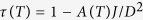. Here, 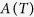 controls the transition and, in our approach, is adopted to reproduce the experimental 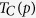 and enable us to predict the phase diagram in absolute units of temperature. The 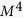 term consists of at least two contributions, (i) the effects of mode-mode interactions 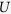 and (ii) the anisotropy contribution 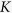, which has been neglected in the effective model in ref^9^. The microscopic parameters 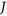, 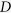 and 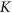 are related to experimental observables in a simple way: 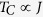, 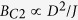 (ref.^29^), while as mentioned previously we assume 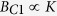.

**Figure 3:**
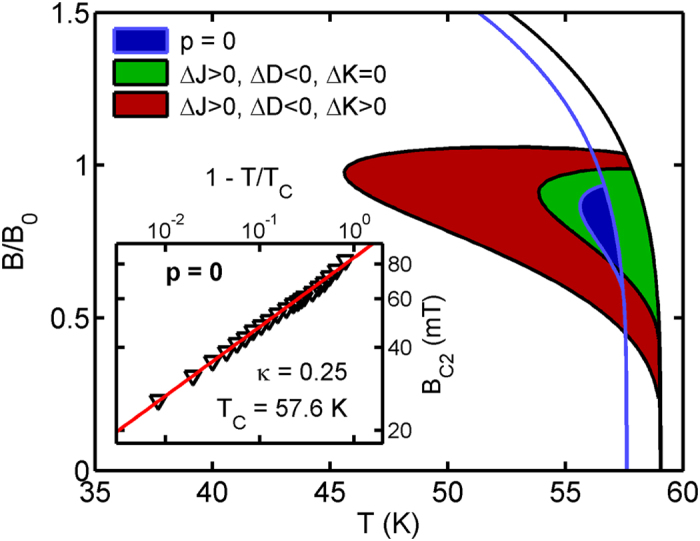
Modeling of the size of the skyrmion pocket using the Ginzburg-Landau approach. The width of the calculated skyrmion pocket (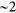 K) for 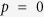 has been used to fix the values of all parameters within the model, see Methods for details. To simulate the behavior at elevated pressures, we change the values of 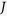, 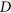 and 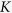 as obtained at 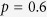 GPa: 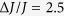
%, 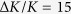
% and 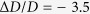
%. The size of the skyrmion pocket is significantly increased only if the change of anisotropy is incorporated in the calculation. Inset: critical behavior of the upper metamagnetic transition between the conical and field-polarized phase 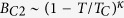.

It has been shown^9^ that when thermal fluctuations around the mean-field solution are included, the skyrmion lattice phase becomes stable close to *T*_*C*_. However, it remained unknown as to the width of the temperature window over which the stabilization can be expected to occur. In order to address this question, first it is necessary to establish the functional relation between the tuning parameter 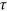 and the thermodynamic temperature 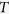. Around the mean-field solution the upper-critical field 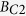 is related to 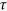 through 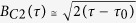, where 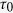 reflects the shift of the transition temperature away from its mean-field value due to the linear effects of fluctuations. In addition, 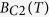 can itself be directly extracted from the experimentally established phase diagram, Fig. 1a. We find that it follows the form of a critical behavior


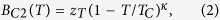


with critical exponent 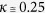, as presented in the inset of Fig. 3. This leads to the simple relation for 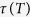



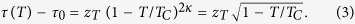


The square-root dependence is preserved across the pressure range investigated in this study.

## Discussion

Now we can proceed with the discussion of the size of the skyrmion pocket in terms of thermodynamic temperature 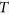. As mentioned above, the inclusion of thermal fluctuations is necessary to stabilize the skyrmion lattice over the conical spin arrangement. However, it has been suggested recently^30^ that the fluctuations close to *T*_*C*_ are strongly interacting, lowering the transition temperature and making it first-order. Within the effective model the reduction in *T*_*C*_ is implemented^9^ by setting the corrections of the order parameter to be smaller than 20%. We keep this approach and in the main panel of Fig. 3 plot the resultant order-disorder boundary with the outline of the skyrmion pocket for different values of 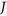, 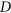 and 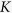. For the zero-pressure case we adjust the coefficients of the model so that the skyrmion pocket is limited to 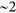 K below *T*_*C*_, as found in the experiment. To explore the effect of pressure we consider the particular case of 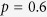 GPa, where the skyrmion pocket is still well defined, and estimate 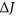, 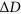 and 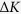 from the experimental data: 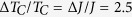 %, 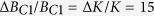 %, while 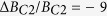 % translates to 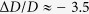 %. It can be shown that the skyrmion pocket grows if the parameter 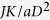 increases, indicating that all of the observed changes contribute positively. If we take into account only 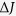 and 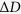 (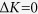) then we obtain a small increase of the skyrmion pocket. However, a more significant growth is obtained when the observed 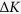 is included, giving 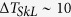 K. The temperature extent is here only indicative since it is known that the Ginzburg-Landau approach is not valid far away from 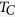, 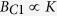 is only a crude approximation which is expected to hold only in a narrow range of parameters and the entropic contribution 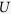 has been neglected. Nevertheless, our analysis demonstrates the importance of anisotropy in stabilizing the skyrmion lattice at temperatures far from the immediate proximity of the ordering temperature. Indeed just 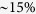 change in effective anisotropy can expand the temperature range of the skyrmion stability by 600%!

The crucial role of anisotropy has also been explored in Monte Carlo simulations^31^ where it has been found that the correct phase diagram can be reproduced if additional anisotropy-compensation terms are added because of boundary effects. On the experimental side, in the recently discovered skyrmion compound GaV_4_S_8_ the orientation of skyrmions is dictated by the magnetic easy axis and not by the direction of magnetic field^32^. At the same time, the relative size of the skyrmion pocket within the phase diagram of this polar magnet is substantially larger than in the family of chiral magnets such as MnSi and 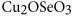.

While the above-presented model is applied as a continuous-field approximation, an important question is what qualitative changes occur with pressure on the level of the unit cell. This is especially important for 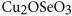 since there are two crystallographically distinct copper sites which couple strongly into tetrahedra with a quantum triplet ground state^29^. These triplets form a trillium lattice which is identical to the lattice of Mn ions in MnSi, and long-range order is then governed by the effective exchange and Dzyaloshinskii-Moriya interactions between the triplets. It follows that the effect of pressure on 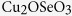 can be two-fold: (i) changing the quantum nature of tetrahedrons and (ii) changing the inter-triplet interactions. In order to probe the former effect, we have performed measurements of the magnetization plateau under pressure. The plateau occurs when the system enters the field polarized state for magnetic fields *B* > 100 mT, see Fig. 2f and inset. It turns out that within the error-bar the observed magnetization plateau does not change, at least for pressures 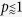 GPa. This means that the tetrahedra remain relatively rigid, while pressure-induced shifts in atomic positions occur dominantly between the tetrahedrons, influencing the effective inter-triplet interactions and justifying the use of the continuous model.

We believe that the results presented in this study and the supporting model clearly demonstrate that although it is the weakest of the energy scales, anisotropy plays a decisive role in stabilization of the skyrmion lattice. Broadening the operational range for the skyrmion lattice and making it more resistant against external fluctuations provides a promising route for future investigations in order to enhance the potential of other skrymion-supporting materials, especially around room temperature^27^, where skyrmion-based applications are foreseen. The deeper theoretical understanding of the various mechanisms leading to skyrmion stability may also guide the rational design of new compounds with favorable characteristics for hosting stable skyrmion phases.

### Experimental conditions

The single crystal sample was prepared by the chemical vapor transport technique. It was aligned by x-ray Laue backscattering and cut along the 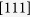 direction with dimensions 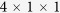
mm^3^. The ac susceptibility measurements were performed using a balanced coils setup. Two detection coils were wound on an inox tube 2 mm in diameter, with centers of the coils 2.5 mm apart. On top of each detection coil a driving coil was made for generation of ac magnetic field. AC current was supplied by a Keithley 6221 current source and the balance of the coils was achieved by changing a resistance in series with each driving coil. The amplitude of ac magnetic field was 0.1 mT. The detection of the ac signal was done by the lock-in amplifier Signal Recovery 7265. The measurements of the phase diagram for each pressure were performed in a slow temperature drift regime, where the sweeping rate has been controlled between 100 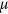
K/s around the skyrmion phase and 700 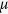
K/s below 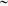
30 K. The magnetic field was generated by a Cryomagnetics 9 T magnet with a sweep rate of 0.1 mT/s (the slowest available on the instrument) between zero and a maximum field value of 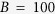 mT. At higher temperatures the upper field value was regularly decreased in order to increase the density of the measured points inside the ordered part of the phase diagram.

The pressure for AC susceptibility study was generated using a non-magnetic piston cylinder pressure-cell with Daphne oil 7373 as a transmitting medium. Pressure was determined using the relative change of the resistance of a manganine wire compared to the zero-pressure case. The magnetization under pressure was measured in a Quantum Design SQUID magnetometer MPMS using a commercial EasyLab pressure cell. The sample used was 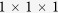 mm^3^ taken from the same batch.

### Ginzburg-Landau phenomenology

The phase diagram is expected to be qualitatively captured by Ginzburg-Landau theory for modulated magnetic structures. For the detailed description of the approach we refer to the ref.^9^,^28^ and just highlight here the main features of the formalism. The calculations are made in the continuous-field approximation justified by the large ratio of the skyrmion radius 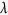 to the lattice constant 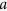. The Ginzburg-Landau functional is taken up to fourth order in magnetization and second order in magnetization gradients, thus including both the Dzyaloshinskii-Moriya interaction and anisotropies. The magnetic structure is determined by minimization of the free energy, where the order parameter is naturally taken as the local magnetization reduced by the average magnetization of the crystal. A mean-field treatment shows the conical phase to be energetically favorable, but the skyrmion phase lies only slightly higher in energy. By including the Gaussian fluctuations of the free energy the skyrmion phase becomes favorable in a certain range of finite magnetic fields. The fluctuations contribute mainly at the short-length scale and are calculated with the cut-off in the momentum space in order of 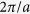. The transition to the paramagnetic state is expected to occur when the fluctuations become significant (around 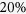 of the mean-field value), which determines the ordered phase boundary through the relation 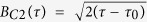. For the calculation of the phase diagram at zero pressure we used the following parameters: 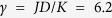, 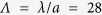, 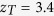 and 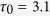. The values of 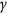 and 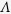 are in good agreement with a recent density functional calculation^29^. The Ginzburg-Landau approach is considered to break down away from 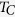 so we leave the field axis normalized to 
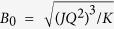
.

## Additional Information

**How to cite this article**: Levatić, I. *et al.* Dramatic pressure-driven enhancement of bulk skyrmion stability. *Sci. Rep.* **6**, 21347; doi: 10.1038/srep21347 (2016).

## References

[b1] Skyrme T. H. R. A unified field theory of mesons and baryons. Nucl. Phys. 31, 556 (1962).

[b2] Sondhi S. L., Karlhede A., Kivelson S. A. & Rezayi E. H. Skyrmions and the crossover from the integer to fractional quantum hall effect at small zeeman energies. Phys. Rev. B 47, 16419 (1993). 10.1103/physrevb.47.16419 10006073

[b3] Brey L., Fertig H. A., Cote R. & MacDonald A. H. Skyrme crystal in a two-dimensional electron gas. Phys. Rev. Lett. 75, 2562 (1995). 10059343 10.1103/PhysRevLett.75.2562

[b4] Ho T. L. Spinor bose condensates in optical traps. Phys. Rev. Lett. 81, 742 (1998).

[b5] Ohmi T. & Machida K. Bose-Einstein condensation with internal degrees of freedom in alkali atom gases. J. Phys. Soc. Jpn. 67, 1822 (1998).

[b6] Khawaja U. A. & Stoof H. Skyrmions in a ferromagnetic Bose-Einstein condensate. Nature 411, 918 (2001). 11418849 10.1038/35082010

[b7] Bogdanov N. & Hubert A. Thermodynamically stable magnetic vortex states in magnetic crystals. J. Magn. Magn. Mater. 138, 255 (1994).

[b8] Rößler U. K., Bogdanov A. N. & Pfleiderer C. Spontaneous skyrmion ground states in magnetic metals. Nature 442, 727 (2012). 10.1038/nature05056 16915285

[b9] Mühlbauer S. *et al.* Skyrmion lattice in a chiral magnet. Science 323, 915 (2009). 19213914 10.1126/science.1166767

[b10] Seki S., Yu X. Z., Ishiwata S. & Tokura Y. Observation of skyrmions in a multiferroic material. Science 336, 198 (2012). 22499941 10.1126/science.1214143

[b11] Jonietz F. *et al.* Spin transfer torques in MnSi at ultralow current densities. Science 330, 1648 (2010). 21164010 10.1126/science.1195709

[b12] White J. S. *et al.* Electric field control of the skyrmion lattice in Cu_2_OSeO_3_. J. Phys. Condens. Matter 24, 432201 (2012). 23032155 10.1088/0953-8984/24/43/432201

[b13] White J. S. *et al.* Electric-field-induced skyrmion distortion and giant lattice rotation in the magnetoelectric insulator Cu_2_OSeO_3_. Phys. Rev. Lett. 113, 107203 (2014). 25238382 10.1103/PhysRevLett.113.107203

[b14] Finazzi M. *et al.* Laser-induced magnetic nanostructures with tunable topological properties. Phys. Rev. Lett. 110, 177205 (2013). 23679767 10.1103/PhysRevLett.110.177205

[b15] Romming N. *et al.* Writing and deleting single magnetic skyrmions. Science 341, 636 (2013). 23929977 10.1126/science.1240573

[b16] Braun H. B. Topological effects in nanomagnetism: from superparamagnetism to chiral quantum solitons. Adv. Phys. 61, 1 (2012).

[b17] Yu X. Z. *et al.* Real-space observation of a two-dimensional skyrmion crystal. Nature 465, 901 (2010). 20559382 10.1038/nature09124

[b18] Butenko A. B., Leonov A. A., Rößler U. K. & Bogdanov A. N. Stabilization of skyrmion textures by uniaxial distortions in noncentrosymmetric cubic helimagnets. Phys. Rev. B 82, 052403 (2010).

[b19] Li Y. *et al.* Robust formation of skyrmions and topological hall effect anomaly in epitaxial thin films of mnsi. Phys. Rev. Lett. 110, 117202 (2013). 25166569 10.1103/PhysRevLett.110.117202

[b20] Tonomura A. *et al.* Real-space observation of skyrmion lattice in helimagnet mnsi thin samples. Nano. Lett. 12, 1673 (2012). 22360155 10.1021/nl300073m

[b21] Peiderer C., Julian S. R. & Lonzarich G. G. Non-fermi liquid nature of the normal state of itinerant-electron ferromagnets. Nature 414, 427 (2001). 11719799 10.1038/35106527

[b22] Doiron-Leyraud N. *et al.* Fermi-liquid breakdown in the paramagnetic phase of a pure metal. Nature 425, 595 (2003). 14534580 10.1038/nature01968

[b23] Pedrazzini P. *et al.* Metallic state in cubic fege beyond its quantum phase transition. Phys. Rev. Lett. 98, 047204 (2007). 17358807 10.1103/PhysRevLett.98.047204

[b24] Huang C. L. *et al.* Observation of a second metastable spin-ordered state in ferrimagnet Cu_2_OSeO_3_. Phys. Rev. B 83, 052402 (2011).

[b25] Sidorov V. A., Petrova A. E., Berdonosov P. S., Dolgikh V. A. & Stishov S. M. Comparative study of helimagnets mnsi and Cu_2_OSeO_3_ at high pressures. Phys. Rev. B 89, 100403 (2014).

[b26] Levatić I., Šurija V., Berger H. & Živković I. Dissipation processes in the insulating skyrmion compound Cu_2_OSeO_3_. Phys. Rev. B 90, 224412 (2014).

[b27] Tokunaga Y. *et al.* A new class of chiral materials hosting magnetic skyrmions beyond room temperature. Nature Communications 6, 7638 (2015). 10.1038/ncomms8638 PMC4506512 26134284

[b28] Izyumov Y. A. Modulated, or long-periodic, magnetic structures of crystals. Usp. Fiz. Nauk 27, 845 (1984).

[b29] Janson O. *et al.* The quantum nature of skyrmions and half-skyrmions in Cu_2_OSeO_3_. Nature Communications 5, 5376 (2014). 10.1038/ncomms6376 25367368

[b30] Janoschek M. *et al.* Fluctuation-induced first-order phase transition in dzyaloshinskii-moriya helimagnets. Phys. Rev. B 87, 134407 (2013).

[b31] Buhrandt S. & Fritz L. Skyrmion lattice phase in three-dimensional chiral magnets from monte carlo simulations. Phys. Rev. B 88, 195137 (2013).

[b32] Kézsmárki I. *et al.* Nel-type skyrmion lattice with confined orientation in the polar magnetic semiconductor GaV_4_S_8_. Nature Mat. 14, 1116 (2015). 10.1038/nmat4402 26343913

